# Quantifying cognition at the bedside: a novel approach combining cognitive symptoms and signs in HIV

**DOI:** 10.1186/s12883-015-0483-1

**Published:** 2015-11-13

**Authors:** Marie-Josée Brouillette, Lesley K. Fellows, Lisa Palladini, Lois Finch, Réjean Thomas, Nancy E. Mayo

**Affiliations:** Department of Psychiatry, Faculty of Medicine, McGill University, Montreal, QC Canada; Chronic Viral Illness Service, McGill University Health Center, Montreal, QC Canada; Research Institute of the McGill University Health Centre, Montreal, QC Canada; Department of Neurology and Neurosurgery, Faculty of Medicine, McGill University, Montreal, QC Canada; Montreal Neurological Institute, McGill University, Montreal, QC Canada; School of Physical and Occupational Therapy, Faculty of Medicine, McGill University, Montreal, QC Canada; Division of Clinical Epidemiology, McGill University Health Center, Montreal, QC Canada; Clinique Médicale l’Actuel, Montreal, QC Canada; McGill University Health Centre, Glen site, 1001 Decarie, D02.4110, Montreal, QC H4A 3J1 Canada

**Keywords:** HIV, Cognitive impairment, Cognitive testing, HAND, Rasch analysis, Practice effect

## Abstract

**Background:**

Up to half of all people with HIV infection have some degree of cognitive impairment. This impairment is typically mild, but nonetheless often disabling. Although early detection of cognitive impairment offers the greatest hope of effective intervention, there are important barriers to this goal in most clinical settings. These include uncertainty about how self-reported cognitive symptoms relate to objective impairments, and the paucity of bedside measurement tools suitable for mild deficits. Clinicians need guidance in interpreting cognitive symptoms in this population, and a brief cognitive measurement tool targeted to mild impairment. We addressed these two problems together here. The objective of this study was to determine the extent to which performance on cognitive tests and self-reported cognitive symptoms form a unidimensional construct.

**Methods:**

Two hundred three HIV+ individuals completed the Montreal Cognitive Assessment, computerized cognitive tasks and a questionnaire eliciting cognitive symptoms. Rasch measurement theory was applied to determine whether patient-reported and performance items could be combined to measure cognition as a unidimensional latent construct.

**Results:**

Performance-based items and cognitive symptoms are arranged hierarchically along the same continuum of cognitive ability, forming a measure with thresholds covering a broad spectrum of ability that has good internal reliability. The cognitive symptoms that fit the measurement model relate to important aspects of everyday life, providing evidence that the identified construct is meaningful.

**Conclusions:**

This finding lays the foundation for a rapid measure of cognitive ability in people with HIV infection that is feasible for routine clinical use, and shows that some cognitive symptoms are systematically related to performance in this population.

## Background

Identification of mild cognitive impairment is increasingly seen as a priority in a variety of disorders that affect the brain. Even very mild deficits may have functional impacts, for example in individuals working in demanding jobs, and there is an increasing focus on intervening at the first signs of decline [1–4]. Interventions aiming to arrest cognitive decline are obviously best delivered as early as possible, to minimize any functional sequelae. There is thus a need for accurate measurement of milder forms of cognitive impairment suitable for routine clinical use. Existing bedside tools (such as the Mini Mental State Examination [MMSE] and the Montreal Cognitive Assessment [MoCA]) are not sensitive to milder impairment, particularly in conditions other than Alzheimer’s disease. Further, these tools produce a score suitable for classifying people as impaired or unimpaired, rather than a measure in the strict sense of the word [5]. Neuropsychological (NP) testing can serve as a measure in this sense, and is sensitive to mild deficits, but is resource-intensive and unavailable in many settings.

The cognitive impairment that is now recognized to occur in 30–50 % of people with HIV infection [2] is a prototypical example of this clinical challenge. Cognitive deficits are typically mild, but can nonetheless affect medication adherence, occupational and social function, and may even accelerate mortality [6–12]. Available screening tools are not sensitive enough to reliably detect these mild impairments, as they were developed to screen for the presence of dementia [13–17]. Milder deficits might be more readily detected by the patients themselves. Patient reports have clear potential advantages: they reflect observations over a longer period of time than bedside testing, and they are, by definition, ecologically valid. However, there is uncertainty about whether patient-reported cognitive difficulties relate to impairment measured on neuropsychological testing, with some suggesting these concerns are more likely to reflect depression [18, 19]. Thus, front line clinicians are poorly equipped to identify those who may need more detailed cognitive assessment, and those who may need treatment of depression or reassurance. Understanding whether patients’ reports of cognitive difficulties are part of the same construct as that measured by performance-based test items is therefore of central importance in improving cognitive assessment in HIV, and perhaps in other conditions with similar patterns of mild cognitive difficulties.

Although cognitive assessment traditionally evaluates several distinct domains (such as memory and executive function), often with the aim of “localizing” deficits to particular brain systems, this approach may be less appropriate in HIV, where deficits appear to arise from widespread brain dysfunction. The conceptualization of HIV-related cognitive impairment in these terms leads to the hypothesis that cognitive ability in HIV can be conceived of as a single latent construct, at least at the level of resolution that can be reasonably achieved by the quick ‘bedside’ cognitive assessment that is needed for routine care.

In support of this hypothesis, several groups, including ours, have demonstrated that items testing a range of cognitive domains (such as specific aspects of memory, attention, and executive function) can be combined to create a calibrated measure of a single latent construct, ‘cognitive ability’, with items ordered by level of difficulty, rather than clustering according to traditional, localizable domains. This has been shown in HIV [20, 21], as well as in other neurological conditions [22–31]. This claim is based on the application of modern psychometric methods, specifically Rasch Measurement Theory (named after Danish statistician Georg Rasch), to cognitive performance data [32]. Rasch Measurement Theory determines the extent to which individual items relating to a latent construct form a unidimensional, linear continuum [33]. As applied to cognition, when the data fit the underlying hierarchical Rasch model, the ordering of the items (e.g. performance on cognitive tests), from easiest to most difficult, provides a method of estimating cognitive ability as a quantity. This approach creates a measure in the strict sense, akin to a “ruler” for the latent construct, producing a quantitative estimate that allows meaningful comparisons of the scores to be made across individuals and within individuals over time [34]. As an illustration of this principle, in two different samples of HIV+ individuals, we have shown that items drawn from the MoCA fit a Rasch model, i.e. could be ordered by level of difficulty, permitting the calculation of a quantitative total score. However, these items alone were too easy to precisely measure the two samples we studied, i.e. many items showed a ceiling effect [20, 21]. In other words, the set of items was poorly targeted to the cognitive ability of the people in the sample.

Here, we build on this work, adding a new sample for more power and asking whether patient-reported items (e.g. “I forget to take my medication”) fit the same Rasch model as cognitive performance items. A positive answer to this question would address three key issues: First, by linking performance on objective tests to reported real life ability, it would be a source of evidence that the cognitive ability construct being assessed has ecological validity. Second, it would enhance the usefulness of the cognitive measure in the clinic by allowing an initial estimation of cognitive ability based on self-report, which would inform the judicious selection of the relevant performance items when required. Finally, it would address whether people with HIV infection without overt dementia have insight into their own cognitive performance, a question of theoretical importance for understanding the neurological and psychological basis of HIV-associated cognitive difficulties. The specific objective of the study was to estimate the extent to which performance-based cognitive test items and self-reported cognitive difficulties form a unidimensional construct in non-demented HIV+ individuals.

## Methods

### Participants

Two different samples were combined for the analysis. The first sample (*n* = 75) was drawn from patients with scheduled appointments at the Chronic Viral Illness Service (CVIS) of the McGill University Health Center from July 2009 to February 2010; this sample has been fully described by Koski et al. [20]. The second sample (*n* = 102) was randomly selected from consecutive patients attending either the CVIS or the Clinique Médicale l’Actuel, a large community clinic serving the HIV+ population in Montreal, between March and September 2012. The second sample was enriched by the targeted recruitment of 26 women.

For both samples, inclusion criteria were: HIV+; aged between 18 and 65 years; and able to communicate in either English or French. Exclusion criteria were: clinically-recognized dementia, history of infection of the central nervous system (CNS) or serious head injury, other neurologic event, active axis 1 psychiatric disorder, substance abuse or use of psychoactive medication likely to substantially interfere with cognition.

### Data collection and ethics, consent and permissions

The local Research Ethics Board (McGill University Health Centre, MUHC, and McGill University) approved the protocols and all subjects provided informed consent (Studies 13-047-BMD and PSY-09-030). A trained research assistant administered all tests and questionnaires in the same session, in either English or French. Clinical and socio-demographic information were collected through a semi-structured interview and chart review.

### Measurement

The selection of items to be tested for fit to the Rasch model was informed by the extensive literature on the cognitive domains typically affected in those with HIV infection [2, 35–38]. Sources of information about cognitive ability consisted of performance on specific cognitive tests and subjects’ answers to questions related to cognition.

Cognitive performance was directly assessed with MoCA items [39] testing the domains of executive function, naming, memory, attention, language, abstraction and orientation. The MoCA was developed as a screening tool for mild cognitive impairment in the older population and, as anticipated, we have previously shown that the items are too easy for our younger patient group that was not selected on the basis of the presence of cognitive difficulties [20, 21]. In order to increase the level of difficulty, we supplemented the MoCA items with more demanding computerized tasks selected from the experimental neuropsychology literature, again focusing on the cognitive processes typically affected in HIV: simple reaction time, verbal and visuospatial working memory was assessed with digit span (forward and backwards) and the Corsi block task (forward and backward) [40], manipulation and updating of verbal and visuospatial material in working memory was assessed with the letter 2-back task [41] and digit span (forward and backwards); choice reaction time and interference control were assessed with the Eriksen flanker task [42]. Standard variables were captured for each task: i.e. reaction time (RT), span, error rates, d’, depending on the task, as in the published work from which they were drawn.

Presence of cognitive difficulties was documented using the 20-item Patient Deficit Questionnaire (PDQ), which assesses self-reported retrospective memory, prospective memory, attention, organization, and planning over the previous 4 weeks. The questionnaire pertains to everyday activities of interest to clinicians, such as adherence to care (e.g. “I forget to take medication” or “I forget medical appointments”) and safety (e.g. “I forget to turn off the stove”), and elicits cognitive difficulties that are frequent among people living with HIV (e.g. “trouble with concentration”). Importantly, the PDQ is brief and can be successfully completed by people with mild to moderate cognitive impairment. The ordinal responses on the 20 items are usually summed to create a total score, with higher scores indicating more difficulty, although here each item was considered individually [43].

Depressive symptoms were documented by the Beck Depression Inventory-II (BDI-II) [44] in the first sample, and the depression sub-scale of the Hospital Anxiety and Depression Scale (HADS-D) [45] in the second sample; traditional cut-offs were applied to define the presence of depressive disorder (BDI-II score ≥ 14, HADS-D score ≥ 8).

### Data analysis

Descriptive statistics were used to characterize the sample. Each item was scored such that a higher value reflected a better cognitive ability. Analyses were conducted according to recommended steps [33] and the RUMM2030 software was used, using the partial-credit model. Rasch analysis proceeded sequentially, fitting the MoCA items first (Item Set one), then adding the computerized items (Item Set two), such that the construct was defined by the performance-based items. Finally, the PDQ items were entered into the model (Item Set 3). Fit to the Rasch model was tested for each sequential Item Set using indices of global fit, fit of individual items and fit of subjects; *p*-values ≥ 0.05 indicate that there is a lack of evidence to reject the underlying hierarchical Rasch Model. This is in contrast to statistical testing of difference where one wants to reject the “null”; when the same test is used as a test of fit, the aim is to not reject the “null”. If the data fit the Rasch model, there is evidence that the items form a measure, in this case of “cognitive ability”, with sufficient mathematical properties. Our sample size of 203 individuals meets criteria for this purpose [46]. A complete description of Rasch Measurement Theory can be found elsewhere [47]. Stability of item calibration across different personal factors (termed Differential Item Functioning or DIF) was tested for age (<45, 45–55, > 55 years), education (<12, ≥12 years), language of test administration and rater. DIF is a feature of an item indicating that it is more or less difficult for certain groups of people; an item with DIF needs to be reworked, rescored or deleted.

Validity of the measure was established in several ways. Criterion validity cannot be ascertained since there is no gold standard *measure* of global cognitive ability (as opposed to a diagnostic classification for HIV-Associated Neurocognitive Disorder (HAND), which is not a measure in the strict sense). Evidence of validity, in Rasch Measurement Theory, is determined by evidence supporting unidimensionality and internal reliability, and, in the usual way, showing construct validity. In keeping with usual practice, construct validity was determined by the ordering of items (with easier item thresholds expected to be lower on the scale of “cognitive ability”), and known-groups analysis (with the assumption that subjects with lower education should have lower scores than those with higher education). Unidimensionality was verified using a principal component analysis (PCA) of the residuals, and internal reliability was measured by the Person Separation Index (PSI) and Cronbach’s alpha.

## Results

Two hundred and three participants were recruited. Table 1 provides the demographic, clinical characteristics, mean MoCA and PDQ scores of the sample. Current CD4 cell count was within the normal range, and 37 % of this sample met criteria for depression based on the standard cut-offs of the screening instruments.

**Table 1:** Characteristics of the sample

Characteristic		Sample (*N*=203)
Demographics		
	Sex (male), *N* (%)	166 (82)
	Age (years), Mean ± SD	48.1± 9.7
	Education (years), Mean ± SD	15 ± 3.8
	Education (years), *N* (%)	
	<12	30 (15)
	≥12	173 (85)
HIV variables		
	Viral load, undetectable, N (%)	182 (90)
	Current CD4 (cells/μL), Mean ± SD	588.8 ± 278.2
	Nadir CD4 (cells/μL), Mean ± SD	273.7 ± 160.1
Clinical variables		
	Depressed, *N* (%)^a^	76 (37)
	PDQ score, Mean ± SD, (0–80)^b^	25 ±13.9
	MoCA score, Mean ± SD, (0–30)	26 ± 2.7
Language of administration, *N* (%)		
	English	51 (25)
	French	152 (75)

^a^HADS-D score ≥ 8 or a BDI-II score ≥ 14

^b^PDQ: Patient Deficit Questionnaire

A total of 62 items were sequentially tested for fit to the Rasch model. Table 2 presents the statistics for global fit, location, and reliability for each item-set tested for fit to the Rasch Model.

**Table 2:** Measurement characteristics of the three models

Model	Items remaining/tested	Fit χ^2^ (p)	Item location (logits)	Person location (logits)	Internal reliability^a^ (PSI)
			Mean (SD)	Mean (SD)	
MoCA alone	23/28	47.9_46df_ (0.39)	0.0 (1.3)	2.3 (0.9)	0.40
MoCA + computerized items	23/23 + 9/14	69.8_60df_ (0.18)	0.0 (1.5)	1.9 (1.1)	0.69
MoCA + computerized items+ PDQ items	21/23 + 8/9 + 9/20	96.3_76df_ (0.06)	0.0 (1.4)	1.9 (0.7)	0.73

^a^Internal reliability or Person Separation Index (PSI) is interpreted as a Cronbach’s alpha

The first item-set tested was the 28 items from the MoCA. Only 23 were retained: 3 were removed because everyone answered them correctly (lion, year, city), and as such they do not contribute to measurement; 2 were deleted because they showed DIF by rater (clock numbers and repetition of the longer sentence), reflecting the difficulty of harmonizing the rating of these items across examiners. Fit to the Rasch model was confirmed for the remaining 23 items with 24 thresholds (*χ*^2^: 47.9; 46df; *p* = 0.39). Item locations by design are standardized at 0 logit and, ideally, have a SD of 1; here the SD was slightly higher at 1.3 logits. Ideally, the mean location of persons should also be 0 with a SD of 1; the mean person location at 2.3 ± 0.9 logits indicates that the items were too easy for the sample tested. The MoCA items alone showed poor internal reliability (PSI = 0.40) for representing the latent construct, cognitive ability.

The second item-set tested included the 23 fitting MoCA items and the 14 items from computerized tasks. After deleting five computerized test items for misfit, fit to the model was verified (*χ*^2^: 69.8; 60df; *p* = 0.18). The mean person location was closer to 0 at 1.9 ± 1.1 logits, and reliability was improved (PSI = 0.69).

Finally, the 20 PDQ items were added to the fitting items from the performance item set. Fitting and deleted items are shown in Table 3. A total of 11 items of the PDQ were deleted for misfit to the model or because they were redundant with other items and did not contribute to measurement. The 9 PDQ items that fit the model reflected difficulties with attention, retrospective memory, prospective memory, and planning. There was DIF by language for one item (PDQ 15); the English and French versions of that item were treated separately. Two additional MoCA items and 1 computerized item were deleted at that stage for misfit (for example, one of the two abstraction items: “watch-ruler”). Measurement characteristics were further improved following inclusion of the PDQ items, resulting in a mean person location of 1.9 ± 0.7 logits with increased reliability (PSI = 0.73). Global fit to the Rasch model was confirmed (*χ*^2^: 96.3; 76df; *p* = 0.06).

**Table 3:** Items on the PDQ tested and retained for the final rasch model

Item number	Retained in model	Item content
1		Lose your train of thought when speaking
2	✓	Have difficulty remembering the names of people, even ones you have met several times
3	✓	Forget what you came into the room for
4		Have trouble getting things organized
5		Have trouble concentrating on what people are saying during a conversation
6		Forget if you had already done something
7	✓	Miss appointments and meetings you had scheduled
8		Have difficulty planning what to do in the day
9	✓	Have trouble concentrating on things like watching a television program or reading a book
10		Forget what you did the night before
11	✓	Forget the date unless you looked it up
12		Have trouble getting started, even if you had a lot of things to do
13		Find your mind drifting
14		Forget what you talked about after a telephone conversation
15	✓	Forget to do things like turn off the stove or turn on your alarm clock
16		Feel like your mind went totally blank
17	✓	Have trouble holding phone numbers in your head, even for a few seconds
18		Forget what you did last week-end
19	✓	Forget to take your medication
20	✓	Have trouble making decisions

The final model thus included 37 items: 28 performance-based items and 9 patient-reported items. These items are shown in Table 4. The computerized tasks are described in more detail elsewhere [20]. Only one person, a recent immigrant from a rural area in a resource-poor country, was not adequately measured by the model.

**Table 4:** Item thresholds of the final model

Location		ITEMS - uncentralised thresholds								
5	|									
	|									
	|	corbw.2								
	|									
	|									
4	|									
	|	digbw.2	m3flc.2							
	|									
	|									
	|									
3	|	fcong.5	Fflu .4							
	|	PDQ 3 .4	PDQ 11.4	Fflu .3						
	|	PDQ 2 .4								
	|	PDQ 17.4								
	|									
2	|	PDQ 15.4	PDQ 20.4							
	|	PDQ 2 .3	PDQ 3 .3							
	|	PDQ 17.2	PDQ 19.4	PDQ 20.3	PDQ15F.1	flan3.2				
	|	PDQ 9 .3	PDQ 9 .4	PDQ 11.3	PDQ 17.3					
	|	mW4 .1	fcong.4							
1	|	PDQ 2 .2	PDQ 7 .4	mW5 .1						
	|									
	|	PDQ 3 .2	PDQ 7 .3	PDQ 11.2	PDQ 20.2					
	|	PDQ 9 .2	PDQ 15.3	PDQ 19.3	mW3 .1	m3flc.1	mW2 .1	mser7.2	digbw.1	seria.1
	|	PDQ 15.2	fcong.3	PDQ15E.1	corbw.1					
0	|	PDQ 7 .2	PDQ 11.1	PDQ 19.2	flan3.1	Fflu .2				
	|	cubes.1	mdigf.1	chand.1						
	|	abstr.1								
	|	PDQ 2 .1	PDQ 17.1							
	|	PDQ 9 .1	name2.1	mdigb.1	trail.1					
−1	|	PDQ 19.1								
	|	Mtrai.1	fcong.2							
	|	date .1								
	|	PDQ 3 .1	PDQ 20.1	mser7.1	tapa .1					
	|	ccont.1	name3.1							
−2	|									
	|									
	|									
	|	day .1	mon .1							
	|									
−3	|									
	|									
	|									
	|	place.1								
	|									
−4	|	PDQ 7 .1	PDQ 15.1	fcong.1						
	|									
	|									
	|									
	|									
−5	|									
	|	Fflu .1								
	|									

Thresholds are listed in order of decreasing difficulty: the more difficult thresholds are shown at the top and the easier ones at the bottom. Items are identified by their item number, followed by their threshold number (Example: PDQ3.2 represents PDQ item 3, 2nd threshold). Abbreviations: ccont: clock contour; chand: clock hand; corbw: corsi backwards; cubes: copying a cube; date: knowing the date; day: knowing the day of the week; digbwA: digits backwards (form A); digbwB: digit backwards (form B); digf: digit forward ; flanAcon: flanker test (form A), congruent reaction time; flanA: flanker test (form A) flanker effect; flanBcon: flanker test (form B) congruent reaction time; Fflu: F fluency; mon: month; ser7A: serial 7 (form A); ser7B: serial 7 (form B); trailA: short trail (form A); trailB: short trail (form B); mW1-mW5: MoCA memory words recall, 1–5; name2-3: name 2^nd^ and 3^rd^ object from MoCA; place: knowing the place; tapa: MoCA tap on letter a

**Fig. 1:**
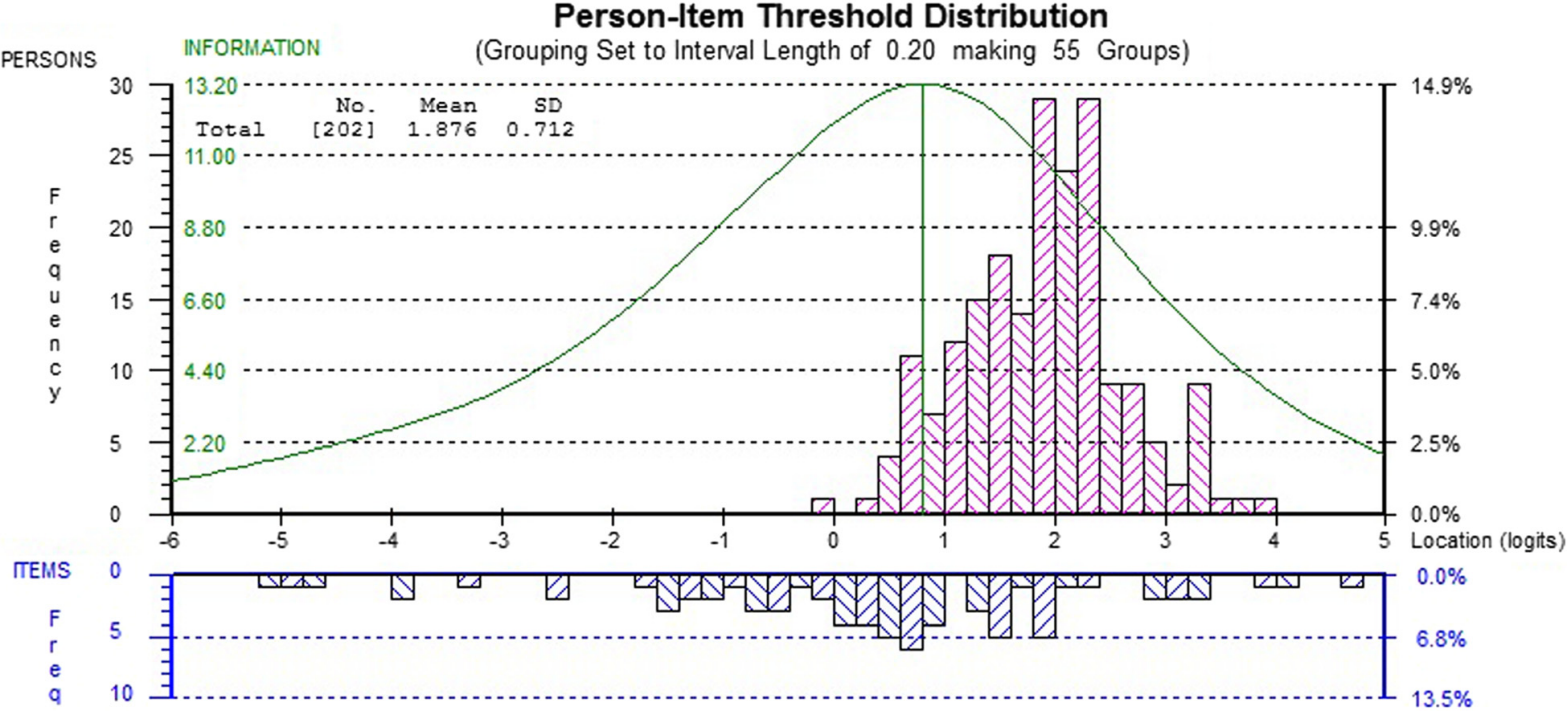
Distribution of individuals with lowest to highest “cognitive ability” (*top*) and of item thresholds (*bottom*). The mean ability of the persons in this sample is 1.9 logits, a value above the mean location of items (0 logit) and with the best measurement precision occurring around 0.8 logits. One person was removed for non-fit. There is no floor or ceiling effect

The frequency of item thresholds and the distribution of individuals with lowest to highest “cognitive ability” are shown in Fig. 1. A measure covering at least −4 to +4 logits (or SD) is considered desirable [48]. In our sample, the range extends from −5.1 to 4.8 logits. Table 4 presents the item thresholds in order of “cognitive ability”. The easiest item threshold is generating 0–4 words on the F fluency test, and the most difficult is repeating backwards a sequence of 7–8 blocks on the Corsi test. PDQ item thresholds, highlighted, are interspersed with the performance-based items, except at the very top and very bottom of the scale; they span a wide range of ability and enrich the item bank at most levels of performance.

Construct validity is supported by the ordering of items as expected, with MoCA items at the lower end of the continuum and computer-administered items at the higher end. Additional evidence for construct validity is provided by model fit, and by the broad spectrum of item threshold coverage. In addition, subjects were grouped as expected: those with ≥ 12 years of education scored significantly higher than those with < 12 years of education (mean: 1.95 ± 0.70 logits versus 1.44 ± 0.62; *p* = 0.0002). Evidence for unidimensionality is provided by the fact that the first PCA from the item-person residuals explained only 10 % of the residual variance. Internal reliability as measured by the PSI was 0.73 and the Cronbach’s alpha was 0.70.

## Discussion

The present study aimed to test whether we could develop a measure of cognitive ability that combines cognitive symptoms and signs. More specifically, we asked whether, among HIV+ individuals without clinically-evident dementia, self-reported items fit the same Rasch model of cognitive ability measured by performance items, and whether self-report contributed additional information to the measurement of that construct. We found that at least some self-report items fit the same construct of “cognitive ability” as measured by performance-based tasks. Rasch Measurement Theory has been previously applied to performance-based cognitive items [20, 31], and has also been applied to develop a measure of physical functioning post-stroke that combines performance (e.g. the two-minute-walk-test) with self-report items (e.g. reported difficulty in doing housework) [48]. Our study shows for the first time that the two sources of information can be combined in the cognitive domain as well, at least in the population we studied: some self-reported difficulties relate in a predictable manner to some performance-based items, a finding that has not been reported before and represents a unique contribution of the application of Rasch measurement theory. We provide mathematical confirmation that items from these two sources of information align, reflecting a single latent construct of “cognitive ability” in people with HIV without overt dementia. The item set covers a broad spectrum of ability with good internal reliability. On a linear continuum, both performance-based and patient-reported items are interspersed across the range of cognitive ability. The most informative model fit parameters indicated that including self-report items improved global model fit.

Evidence for construct validity also emerged. The data fit the Rasch model [49] and the item-person hierarchy is reliable. Fit to model is judged both statistically and theoretically, with global fit p-value only one of several criteria; targeting of the items to the people is an important consideration and as the computerized and self-report items were added, targeting and reliability improved without rejecting the model. In addition, a known-groups analysis confirmed that, as expected, participants with less education had lower scores. We could not assess criterion validity, as there is no gold standard measure of cognitive ability in HIV. While consensus criteria do exist for making a diagnosis of HAND [50], measuring cognition is distinct from diagnostic classification. As applied here, measurement aims to describe cognitive ability in terms of a quasi-continuous “amount” across the whole range of ability. In contrast, a HAND diagnosis involves categorizing people into groups based on degree of impairment in specific cognitive domains. While in principle these two approaches could align, we have demonstrated in separate work that, in fact, there is poor concordance between *measurement* of cognitive ability (based on MoCA items) and *diagnosis* of HAND [21]. The present study did not aim to examine the relationship between these two approaches, instead focusing on a measurement gap, the lack of a measure of cognitive ability with a legitimate total score, as the critical initial step.

Including self-report in a cognitive measure has several advantages. First, linking performance on objective tests to reported real life ability is a source of evidence that the cognitive construct being assessed has ecological validity. The specific patient-reported items that fit the model assess important real-life activities such as forgetting to take medication, to attend medical appointments, or to turn off the stove, or having difficulty making decisions. Future work could provide further evidence of ecological validity through direct observation of real life function, although there is already evidence linking self-reported cognitive complaints with objective assessment of everyday function in HIV [51, 52]. Second, as an initial step in the measurement of cognition, self-reports are easy to obtain and can inform the selection of performance items that are most likely to be informative, making the assessment as brief as possible by avoiding items that are too easy or too difficult for the specific person. We show that specific self-report items can be integrated directly into the measurement of cognitive ability in a given individual. Rasch-developed measures lend themselves readily to this adaptive administration: the ladder-like quantification of the items means that only performance on those items around the person’s specific “rung” need to be evaluated, saving time without sacrificing precision. This approach also provides a flexible foundation for further optimizing the set of items, as additional items can be easily tested and related to the core set.

Our study has limitations. Although the specific set of items here defines a unidimensional construct of cognitive ability, it is by no means the definitive set. While the characteristics of the measure generated in this study are promising, more items are required at the higher end of the “cognitive ability” spectrum in order to further improve the precision of the measure; this will be particularly important to monitor cognition in people with high baseline cognitive function.

The patient-reported item set studied here was not originally developed for use in HIV. While the items address a range of common cognitive complaints, we do not know whether these particular items are optimal for eliciting cognitive difficulties in people living with HIV. Development of HIV-specific items may add measurement precision. Further study is also required to revalidate the scoring options of the items, establish stability over time, and assure the performance of the measure in varied samples. In addition, we cannot assume that the hierarchy of items would hold in clinical populations that are different from the one in which it was developed, for example in people with other neurological disorders or frank HIV-associated dementia. Poor performance in the absence of awareness of limitations would presumably change the interplay between patient-reported and performance-based items, and so would affect the hierarchy and fit. However, the greatest unmet clinical need at this point is for early identification of mild cognitive impairment; the present study shows that assessment of symptoms and signs can be combined in this context.

Lack of self-reported difficulties in spite of the presence of profound cognitive impairment is a feature of dementia associated with HIV infection and other neurological conditions. Arriving at a value for cognitive ability based exclusively on self-report would be inaccurate under those conditions. However, in the non-demented range of cognitive ability tested here, we confirmed that self-reports on specific questions about cognitive performance do provide useful information, and relate in an orderly fashion to performance on objective tests. Importantly, deviation from that orderly relationship in an individual’s responses could be used to detect potential loss of insight.

The sample studied here had a relatively high prevalence of at least mild depressive symptoms. This is an advantage in that it demonstrates that patient-reports and cognitive performance can be aligned on a common scale even in the presence of depression. However, further work would be needed to confirm that the item hierarchy holds in larger samples, and in samples where the rates of depression differ markedly from the one studied here. The recruitment strategies we used likely yielded a sample with minimal selection bias, fairly representative of our clinical population, arguing that the measure is widely applicable.

## Conclusions

In summary, we found that cognition in HIV+ individuals without overt dementia can be conceived of as a unitary latent construct, which can be assessed by ascertaining cognitive symptoms and signs. The final set of 37 items that fit the model can be administered and scored in less than 30 min, and provides a useful starting point for a brief measure of cognition suitable for everyday clinical use. With further refinement, this will equip clinicians and researchers alike with a method for measuring cognition in people with HIV. Further work can show how it relates to existing diagnostic classifications and to real world function. Given that mild memory, executive and attentional difficulties are a feature of several common neurological and medical disorders, this approach may also prove to be useful for other conditions where clinicians face similar cognitive assessment challenges. The present study is an important first step in addressing recent calls for accessible, efficient, high quality cognitive assessment tools feasible for a wide range of clinical settings.

## Abbreviations

MMSE: Mini Mental State Examination
MoCA: Montreal Cognitive Assessment
NP: Neuropsychological
CVIS: Chronic Viral Illness Service
CNS: Central Nervous System
RT: Reaction Time
PDQ: Patient Deficit Questionnaire
HADS: Hospital Anxiety and Depression Scale
DIF: Differential Item Functioning
HAND: HIV-Associated Neurocognitive Disorder
PCA: Principal Component Analysis
PSI: Person Separation Index

## References

[1] Cysique LA, Letendre SL, Ake C, Jin H, Franklin DR, Gupta S, Shi C, Yu X, Wu Z, Abramson IS (2010). Incidence and nature of cognitive decline over 1 year among HIV-infected former plasma donors in China. AIDS.

[2] Heaton RK, Clifford DB, Franklin DR, Woods SP, Ake C, Vaida F, Ellis RJ, Letendre SL, Marcotte TD, Atkinson JH (2010). HIV-associated neurocognitive disorders persist in the era of potent antiretroviral therapy: CHARTER Study. Neurology.

[3] Schindler RJ (2005). Dementia with cerebrovascular disease: the benefits of early treatment. Eur J Neurol.

[4] Lista S, Dubois B, Hampel H (2015). Paths to Alzheimer's disease prevention: from modifiable risk factors to biomarker enrichment strategies. J Nutr Health Aging.

[5] Grimby G, Tennant A, Tesio L (2012). The use of raw scores from ordinal scales: time to end malpractice?. J Rehabil Med.

[6] Jia H, Uphold CR, Wu S, Reid K, Findley K, Duncan PW (2004). Health-related quality of life among men with HIV infection: effects of social support, coping, and depression. AIDS Patient Care STDS.

[7] Letendre SL, McCutchan JA, Childers ME, Woods SP, Lazzaretto D, Heaton RK, Grant I, Ellis RJ (2004). Enhancing antiretroviral therapy for human immunodeficiency virus cognitive disorders. Ann Neurol.

[8] Power C, Boisse L, Rourke S, Gill MJ (2009). NeuroAIDS: an evolving epidemic. Can J Neurol Sci.

[9] Price RW, Yiannoutsos CT, Clifford DB, Zaborski L, Tselis A, Sidtis JJ, Cohen B, Hall CD, Erice A, Henry K (1999). Neurological outcomes in late HIV infection: adverse impact of neurological impairment on survival and protective effect of antiviral therapy. AIDS Clinical Trial Group and Neurological AIDS Research Consortium study team. AIDS.

[10] Tozzi V, Balestra P, Galgani S, Murri R, Bellagamba R, Narciso P, Antinori A, Giulianelli M, Tosi G, Costa M (2003). Neurocognitive performance and quality of life in patients with HIV infection. AIDS Res Hum Retroviruses.

[11] Vivithanaporn P, Heo G, Gamble J, Krentz HB, Hoke A, Gill MJ, Power C (2010). Neurologic disease burden in treated HIV/AIDS predicts survival: a population-based study. Neurology.

[12] Ellis RJ, Rosario D, Clifford DB, McArthur JC, Simpson D, Alexander T, Gelman BB, Vaida F, Collier A, Marra CM (2010). Continued high prevalence and adverse clinical impact of human immunodeficiency virus-associated sensory neuropathy in the era of combination antiretroviral therapy: the CHARTER Study. Arch Neurol.

[13] Valcour V, Paul R, Chiao S, Wendelken LA, Miller B (2011). Screening for cognitive impairment in human immunodeficiency virus. Clin Infect Dis.

[14] Kamminga J, Cysique LA, Lu G, Batchelor J, Brew BJ (2013). Validity of cognitive screens for HIV-associated neurocognitive disorder: a systematic review and an informed screen selection guide. Curr HIV/AIDS Rep.

[15] Power C, Selnes OA, Grim JA, McArthur JC (1995). HIV Dementia Scale: a rapid screening test. J Acquir Immune Defic Syndr Hum Retrovirol.

[16] Zipursky AR, Gogolishvili D, Rueda S, Brunetta J, Carvalhal A, McCombe JA, Gill MJ, Rachlis A, Rosenes R, Arbess G (2013). Evaluation of brief screening tools for neurocognitive impairment in HIV/AIDS: a systematic review of the literature. AIDS.

[17] Mind Exchange Working Group (2013). Assessment, diagnosis, and treatment of HIV-associated neurocognitive disorder: a consensus report of the mind exchange program. Clin Infect Dis.

[18] Cysique LA, Maruff P, Darby D, Brew BJ (2006). The assessment of cognitive function in advanced HIV-1 infection and AIDS dementia complex using a new computerised cognitive test battery. Arch Clin Neuropsychol.

[19] Thein HH, Maruff P, Krahn MD, Kaldor JM, Koorey DJ, Brew BJ, Dore GJ (2007). Improved cognitive function as a consequence of hepatitis C virus treatment. HIV Med.

[20] Koski L, Brouillette MJ, Lalonde R, Hello B, Wong E, Tsuchida A, Fellows L (2011). Computerized testing augments pencil-and-paper tasks in measuring HIV-associated mild cognitive impairment. HIV Med.

[21] Brouillette MJ, Mayo N, Fellows LK, Lebedeva E, Higgins J, Overton ET, Ances BM, Koski L (2015). A better screening tool for HIV-associated neurocognitive disorders: is it what clinicians need?. AIDS.

[22] Koski L, Xie H, Finch L (2009). Measuring cognition in a geriatric outpatient clinic: Rasch analysis of the Montreal Cognitive Assessment. J Geriatr Psychiatry Neurol.

[23] Koski L, Xie H, Konsztowicz S (2011). Improving precision in the quantification of cognition using the Montreal Cognitive Assessment and the Mini-Mental State Examination. Int Psychogeriatr.

[24] Larson EB, Heinemann AW (2010). Rasch analysis of the Executive Interview (The EXIT-25) and introduction of an abridged version (The Quick EXIT). Arch Phys Med Rehabil.

[25] Forjaz MJ, Ayala A, Rodriguez-Blazquez C, Frades-Payo B, Martinez-Martin P (2010). Assessing autonomic symptoms of Parkinson's disease with the SCOPA-AUT: a new perspective from Rasch analysis. Eur J Neurol.

[26] Forjaz MJ, Frades-Payo B, Rodriguez-Blazquez C, Ayala A, Martinez-Martin P (2010). Should the SCOPA-COG be modified? A Rasch analysis perspective. Eur J Neurol.

[27] Posner HB, Cano S, Carrillo MC, Selnes O, Stern Y, Thomas RG, Zajicek J, Hobart J (2013). Establishing the psychometric underpinning of cognition measures for clinical trials of Alzheimer's disease and its precursors: a new approach. Alzheimers Dement.

[28] Bode RK, Heinemann AW, Semik P (2000). Measurement properties of the Galveston Orientation and Amnesia Test (GOAT) and improvement patterns during inpatient rehabilitation. J Head Trauma Rehabil.

[29] Bode RK, Heinemann AW, Kozlowski AJ, Pretz CR (2014). Self-scoring templates for motor and cognitive subscales of the FIM instrument for persons with spinal cord injury. Arch Phys Med Rehabil.

[30] Freitas S, Prieto G, Simoes MR, Santana I (2014). Psychometric properties of the Montreal Cognitive Assessment (MoCA): an analysis using the Rasch model. Clin Neuropsychol.

[31] Hobart J, Cano S, Posner H, Selnes O, Stern Y, Thomas R, Zajicek J (2013). Putting the Alzheimer's cognitive test to the test II: Rasch Measurement Theory. Alzheimers Dement.

[32] Andrich D (2011). Rating scales and Rasch measurement. Expert Rev Pharmacoecon Outcomes Res.

[33] Pallant JF, Tennant A (2007). An introduction to the Rasch measurement model: an example using the Hospital Anxiety and Depression Scale (HADS). Br J Clin Psychol.

[34] Wright B, Masters GN (1982). Rating Scale Analysis.

[35] Antinori A, Perno CF, Giancola ML, Forbici F, Ippolito G, Hoetelmans RM, Piscitelli SC (2005). Efficacy of cerebrospinal fluid (CSF)-penetrating antiretroviral drugs against HIV in the neurological compartment: different patterns of phenotypic resistance in CSF and plasma. Clin Infect Dis.

[36] Reger M, Welsh R, Razani J, Martin DJ, Boone KB (2002). A meta-analysis of the neuropsychological sequelae of HIV infection. J Int Neuropsychol Soc.

[37] Woods SP, Moore DJ, Weber E, Grant I (2009). Cognitive neuropsychology of HIV-associated neurocognitive disorders. Neuropsychol Rev.

[38] Heaton RK, Cysique LA, Jin H, Shi C, Yu X, Letendre S, Franklin DR, Ake C, Vigil O, Atkinson JH (2008). Neurobehavioral effects of human immunodeficiency virus infection among former plasma donors in rural China. J Neurovirol.

[39] Nasreddine ZS, Phillips NA, Bedirian V, Charbonneau S, Whitehead V, Collin I, Cummings JL, Chertkow H (2005). The Montreal Cognitive Assessment, MoCA: a brief screening tool for mild cognitive impairment. J Am Geriatr Soc.

[40] Robbins TW, James M, Owen AM, Sahakian BJ, Lawrence AD, McInnes L, Rabbitt PM (1998). A study of performance on tests from the CANTAB battery sensitive to frontal lobe dysfunction in a large sample of normal volunteers: implications for theories of executive functioning and cognitive aging. Cambridge Neuropsychological Test Automated Battery. J Int Neuropsychol Soc.

[41] Tsuchida A, Fellows LK (2009). Lesion evidence that two distinct regions within prefrontal cortex are critical for n-back performance in humans. J Cogn Neurosci.

[42] Modirrousta M, Fellows LK (2008). Dorsal medial prefrontal cortex plays a necessary role in rapid error prediction in humans. J Neurosci.

[43] Sullivan JJL, Edgley K, Dehoux E (1990). A survey of multiple sclerosis. Part 1: Perceived cognitive problems and compensatory straategy use. Can J Reha.

[44] Beck AT, Steer RA, Brown GK (1996). Beck Depression Inventory.

[45] Zigmond AS, Snaith RP (1983). The hospital anxiety and depression scale. Acta Psychiatr Scand.

[46] Linacre JM (2002). Optimizing rating scale category effectiveness. J Appl Meas.

[47] Andrich D (1988). Rasch Models for measurement.

[48] Finch LE, Higgins J, Wood-Dauphinee S, Mayo NE (2008). A measure of early physical functioning (EPF) post-stroke. J Rehabil Med.

[49] Smith EV (2001). Evidence for the reliability of measures and validity of measure interpretation: a Rasch measurement perspective. J Appl Meas.

[50] Antinori A, Arendt G, Becker JT, Brew BJ, Byrd DA, Cherner M, Clifford DB, Cinque P, Epstein LG, Goodkin K (2007). Updated research nosology for HIV-associated neurocognitive disorders. Neurology.

[51] Heaton RK, Marcotte TD, Mindt MR, Sadek J, Moore DJ, Bentley H, McCutchan JA, Reicks C, Grant I (2004). The impact of HIV-associated neuropsychological impairment on everyday functioning. J Int Neuropsychol Soc.

[52] Carter SL, Rourke SB, Murji S, Shore D, Rourke BP (2003). Cognitive complaints, depression, medical symptoms, and their association with neuropsychological functioning in HIV infection: a structural equation model analysis. Neuropsychology.

